# A review of experimental studies of hydrogen as a new therapeutic agent in emergency and critical care medicine

**DOI:** 10.1186/2045-9912-4-17

**Published:** 2014-11-08

**Authors:** Meihua Shen, Hongying Zhang, Congjun Yu, Fan Wang, Xuejun Sun

**Affiliations:** Department of Emergency, Shanghai Provincial Crops Hospital, Chinese People’s Armed Police Forces, 831HongXu Road, Shanghai, 201103 PR China; Department of Quality Management, General Hospital, Chinese Armed Police Force, 69YongDing Road, Beijing, 100039 PR China; Department of Medical Abministration, General Hospital, Chinese Armed Police Force, 69YongDing Road, Beijing, 100039 PR China; Department of Diving Medicine, Faculty of Naval Medicine, Second Military Medical University, 800XiangYin Road, Shanghai, 200433 PR China

**Keywords:** Hydrogen, Reactive oxygen species, Antioxidant, Emergency, Critical care medicine

## Abstract

Hydrogen is the most abundant chemical element in the Universe, but is seldom regarded as a therapeutic agent. Recent evidence has shown that hydrogen is a potent antioxidative, antiapoptotic and anti-inflammatory agent and so may have potential medical applications in cells, tissues and organs. There are several methods to administer hydrogen, such as inhalation of hydrogen gas, aerosol inhalation of a hydrogen-rich solution, drinking hydrogen dissolved in water, injecting hydrogen-rich saline (HRS) and taking a hydrogen bath. Drinking hydrogen solution (saline/pure water/other solutions saturated with hydrogen) may be more practical in daily life and more suitable for daily consumption. This review summarizes the findings of recent studies on the use of hydrogen in emergency and critical care medicine using different disease models.

## Introduction

**Figure 1:**
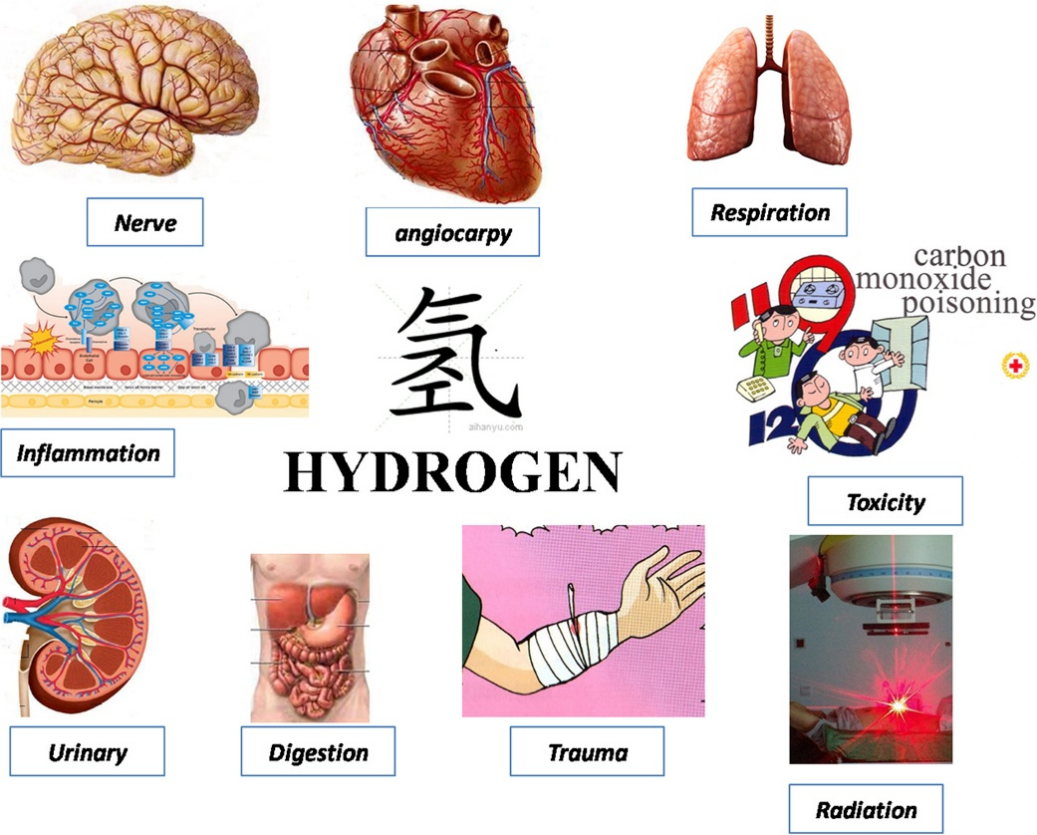
**Summary of potential of various preventive and therapeutic effects of hydrogen in emergency and critical care medicine using different disease models.**

Hydrogen is the lightest element in the Periodic Table and the most abundant chemical substance in the Universe. Most hydrogen is employed near its production site, with the two largest uses being fossil fuel processing and ammonia production, mostly for the fertilizer market. Hydrogen is seldom regarded as an important agent in medical use, especially as a therapeutic gas. However, in July 2007 researchers from the Japan Medical University Institute of Geriatrics reported that inhaled hydrogen gas has antioxidant and antiapoptotic properties that protect the brain against ischemia–reperfusion (I/R) injury and stroke by selectively reducing hydroxyl radicals (·OH) and ONOO^−^ in cell-free systems [1]. This study aroused interest worldwide and scientists have explored the therapeutic value of hydrogen in many disease models. Accumulating evidence suggests that hydrogen can protect various cells, tissues and organs against oxidative injury [2].This review focuses on the findings of recent studies of the effects of hydrogen in different disease models in emergency and critical care medicine, as shown in Figure 1. The possible mechanisms involved in its protective effects are summarized.

## Review

### Hydrogen therapy in the nervous system

It was first reported in 2007 that inhaled hydrogen gas has antioxidant and antiapoptotic properties that protect the brain against I/R injury and stroke. In an in vitro study, researchers demonstrated that hydrogen functions as a scavenger of ·OH. Then in a neonatal hypoxia–ischemia rat model, we found that 2% hydrogen gas or HRS therapy reduced apoptosis [3, 4]. However, another group has reported that 2.9% hydrogen gas therapy does not ameliorate moderate-to-severe ischemic damage in a neonatal hypoxia–ischemia rat model [5], although they did find that hydrogen gas reduced infarction and hemorrhage and improved neurologic function in a rat model of middle cerebral artery occlusion. Inhalation of hydrogen gas ameliorated intracerebral hemorrhage in mice [6], and hydrogen saline protected against brain injury from experimental subarachnoid hemorrhage [7] and spinal cord I/R injury [8]. It has been reported that HRS attenuated neuronal I/R injury by preserving mitochondrial function [9]. Hong and colleagues concluded that hydrogen can protect against neurologic damage and apoptosis early in brain injury following subarachnoid hemorrhage through the Akt/hGSK3β signaling pathway [10]. Also, we found that hydrogen saline decreased 8-hydroxyl-2'-deoxyguanosine (8-OHdG), reduced malondialdehyde (MDA), interleukin-1β (IL-1β) and tumor necrosis factor alpha (TNF-α) and suppressed caspase-3 activity in the ischemic brain [11]. Hyperglycemia is one of the major factors contributing to hemorrhage after ischemic stroke. Chen et al. found that the protective effect of hydrogen in the rat brain is accompanied by a reduction in oxidative stress and blood glucose levels after dextrose injection [12]. In addition, hydrogen-rich pure water has been reported to prevent superoxide formation in brain slices from vitamin C depleted SMP30/GNL-knockout mice during hypoxia–re-oxygenation [13]. In a model of perinatal asphyxia in newborn pigs, ventilation with 2.1% hydrogen-supplemented room air significantly preserved cerebrovascular reactivity to hypercapnia and reduced neuronal injury induced by asphyxia–re-ventilation [14]. Interestingly, our group found that lactulose, which is used in the treatment of constipation and hepatic encephalopathy, ameliorated cerebral I/R injury by inducing hydrogen [15]. Finally, it has been reported that drinking hydrogen-rich water ameliorated cognitive impairment in mice with accelerated senescence [16].

### Hydrogen therapy in the respiratory system

The role of oxidative stress is well appreciated in the development of acute lung injury (ALI). Oxidative stress in ALI is believed to be initiated by products of activated lung macrophages and infiltrating neutrophils and propagates rapidly to lung epithelial and endothelial cells, leading to tissue damage and organ dysfunction. Severe burns with delayed resuscitation caused rapid lung edema and impaired oxygenation in rats, but this was ameliorated by intraperitoneal administration of HRS [17]. In a rat model of lung injury induced by intestinal I/R, HRS treatment decreased neutrophil infiltration, lipid membrane peroxidation, nuclear factor kappa B (NF-κB) activation and levels of the proinflammatory cytokines IL-1β and TNF-α in lung tissues compared with saline treatment, thereby attenuating lung injury [18]. Xie and colleagues demonstrated that molecular hydrogen treatment ameliorated lipopolysaccharide (LPS)-induced ALI by reducing lung inflammation and apoptosis, which may be associated with decreased NF-κB activity [19]. Also, inhalation of hydrogen gas reduces hyperoxic lung injury in vivo [20]. Combined with fluid resuscitation, hydrogen inhalation attenuated lung and intestinal injury [21]. Hydrogen saline reduced airway remodeling via inactivation of NF-κB in a murine model of asthma [22].

### Hydrogen therapy in the cardiovascular system

Emergency doctors frequently encounter acute myocardial infarction and are alert to this condition. The accelerated generation of reactive oxygen species (ROS) by reperfusion of the ischemic myocardium is a potential mediator of reperfusion injury. It has been reported that inhalation of 2% hydrogen gas rapidly increased the regional concentration of hydrogen in the area at risk of myocardial infarction before coronary blood flow was re-established in the occluded artery and alleviated I/R injury at the time of recanalization of the coronary artery [23]. Another study using the same model showed that HRS treatment attenuated regional myocardial I/R-induced cell apoptosis, as demonstrated by significant improvement in heart function parameters [24]. Furthermore, HRS decreased oxidative stress and inflammation in the area at risk in rat hearts [25]. Combination therapy with hydrogen and carbon monoxide in a syngeneic heterotopic heart transplantation model showed enhanced therapeutic efficacy via both antioxidant and anti-inflammatory mechanisms [26]. Breathing hydrogen gas plus nitric oxide reduced I/R injury in murine heart [27]. Hydrogen-containing saline protected against doxorubicin-induced heart failure [28]. There are five types of shock, among which hemorrhagic shock predominates in emergency department. Uncontrolled hemorrhagic shock can cause organ hypoperfusion, tissue ischemia and hypoxia, inflammatory cytokine release and the generation of excess oxygen-derived free radicals, all of which can result in multiple organ dysfunction. Du and colleagues reported that HRS protected against uncontrolled hemorrhagic shock [29]. Hydrogen can also reduce cerebral I/R injury and improve the prognosis of cardiopulmonary cerebral resuscitation after cardiac arrest [30].

### Hydrogen therapy in the digestive system

Fukuda et al. found that inhalation of 2% hydrogen gas can suppress hepatic injury caused by warm I/R by reducing oxidative stress [31]. Recently, we reported protective effects of HRS on liver I/R injury due to reduced oxidative stress and high mobility group box 1 (HMGB1) release [32]. In another study, intestinal damage was detected under a microscope and assessed using the Chiu scoring system after I/R injury. Serum diamine oxidase activity, tissue MDA and myeloperoxidase (MPO) activity, and serum TNF-α, IL-1β and IL-6 levels were all increased significantly by I/R injury. HRS reduced these tissue injury markers and relieved the morphologic intestinal injury [33]. Simultaneously, HRS treatment significantly attenuated the severity of intestinal I/R injury by inhibiting apoptosis and promoting enterocyte proliferation, limiting neutrophil infiltration and lipid oxidation, and ameliorating the decreased contractility response to potassium chloride [34]. Moreover, HRS treatment also significantly attenuates the severity of L-arginine-induced acute pancreatitis in rats by inhibiting oxidative stress, apoptosis and NF-κB activation and promoting acinar cell proliferation [35]. Sun found that acute liver injury, hepatic cirrhosis and hepatocyte proliferation in experimental liver injury were reduced by HRS through the quenching of detrimental ROS [36]. Moreover, HRS was shown to attenuate liver damage induced by obstructive jaundice or endotoxin [37, 38] and to protect against necrotizing enterocolitis in neonatal rats [39] and acute peritonitis [40].

### Hydrogen therapy in the urinary system

Renal I/R injury, including transplantation, surgical revascularization of the renal artery, shock, partial nephrectomy and the treatment of suprarenal aortic aneurysms, is a common finding in clinical settings. Mediated by ROS, I/R is the primary cause of acute kidney injury, particularly in patients hospitalized in intensive care units. Shingu et al. reported that, after I/R injury, serum 8-OHdG levels were significantly increased; histologic analysis revealed interstitial congestion, edema, inflammation and hemorrhage in renal tissue. HRS reversed these changes and relieved mitochondrial morphologic renal injury [41]. In our study, a rat model of renal I/R injury was induced by 45 min of occlusion of both renal pedicles followed by 24 h of reperfusion. We found that HRS attenuated the renal I/R injury, possibly by reduction of oxidative stress and inflammation [42]. In February 2014, it was reported that inhalation of hydrogen (2.5%) markedly reduced levels of serum BUN, CREA and MDA, and thus could attenuate renal I/R injury (I/R of the left renal pedicle following right nephrectomy) in rats [43].

### Hydrogen therapy and trauma

It has been reported that administration of HRS reduced acute spinal cord contusion injury by decreasing the number of apoptotic cells, suppressing oxidative stress, increasing the release of brain derived neurotrophic factor and improving locomotor function [44]. Inhalation of 2% hydrogen significantly attenuated traumatic brain injury in rats [45]. Hydrogen treatment before irradiation significantly inhibited ionizing radiation-induced injury in human lymphocyte AHH-1 cells and protected the gastrointestinal endothelium of mice against radiation-induced injury [46]. Recently, it was shown that irrigation of the cornea with isotonic hydrogen solution significantly reduced angiogenesis after alkali burn injury, and hydrogen down-regulated ROS production by the cornea, NF-κB phosphorylation and levels of vascular endothelial growth factor and monocyte chemoattractant protein-1 [47]. Drinking water protects against neurodegenerative changes induced by traumatic brain injury [48].

### Hydrogen therapy and inflammation

Some studies have reported the effect of hydrogen treatment in models of inflammation. First, concanavalin A (ConA)-induced liver inflammation in mice was decreased following the administration of hydrogen-producing bacteria or hydrogen water. Second, the introduction of hydrogen down-regulated the in vitro production of both TNF-α and interferon-γ by ConA-stimulated spleen lymphocytes [49]. Thirdly, hydrogen-saturated water has been shown to prevent the development of dextran sodium sulfate-induced colitis in mice, this effect most likely being due to suppression by hydrogen of macrophage activation in response to luminal bacterial antigens such as LPS [50]. Furthermore, the findings of Zhen et al. indicate that HRS had an anti-inflammatory effect in both LPS-activated macrophages and paw edema models [51]. In a mouse model of systematic inflammation, hydrogen inhalation significantly improved the survival rate of septic mice in a concentration and time dependent manner [52]. Treatment of mice with 2% hydrogen had beneficial effects on sepsis and sepsis associated organ damage, as demonstrated by decreased levels of oxidative products, increased antioxidant enzyme activity and reduced levels of (HMGB1) in serum and tissue. Furthermore, 2% hydrogen treatment has been reported to protect mice against multiple organ damage in a zymosan-induced generalized model of inflammation [53]. Some studies have found that hyperoxia may be beneficial in sepsis. However, the clinical use of hyperoxia is hindered by concerns that it could exacerbate organ injury by increasing free radical formation. Xie et al. found that combination therapy with hydrogen gas and hyperoxia has enhanced therapeutic efficacy via both antioxidant and anti-inflammatory mechanisms and might potentially be a clinically feasible approach for sepsis [54]. The same team reported in 2014 that inhalation of hydrogen gas attenuates brain injury resulting from sepsis [55].

### Hydrogen therapy and toxicity

Hydrogen therapy can attenuate many kinds of cell damage resulting from drugs or chemicals. The anticancer drug cisplatin is widely employed in the treatment of many types of tumor; however, its use is limited by nephrotoxicity due to oxidative stress. Japanese researchers reported that inhalation of 1% hydrogen gas or drinking hydrogen water ad libitum alleviated this toxicity by reducing oxidative stress [56]. Also, hydrogen reduced mortality and body weight loss and improved metamorphosis accompanying decreased apoptosis in the kidney, but did not impair the antitumor activity of cisplatin against cancer cell lines in vitro or in tumor-bearing mice in vivo. The protective effect of HRS against cisplatin-induced nephrotoxicity was verified in rats using dynamic contrast enhanced computed tomography [57]. In a model of antimycin A-induced auditory hair cell damage, incubation of hair cells in a hydrogen-saturated medium significantly reduced the generation of ROS and subsequent lipid peroxidation in auditory epithelia, leading to increased hair cell survival [58]. Hydrogen gas has also been reported to protect against the morphologic and functional vestibular hair cell damage induced by ROS [59]. Exposure to high concentrations of oxygen may lead to ALI. Zheng et al. [60] found that saturated hydrogen saline alleviated hyperoxia-induced pulmonary injury, which was partly responsible for the inhibition of oxidative damage. It was also found that HRS ameliorated hyperoxia-induced ALI by reducing oxidative stress and inflammatory cascades in lung tissue [61]. Increased production of ROS is crucial in the pathogenesis of carbon monoxide poisoning. In our research, we found that hydrogen provides neural protection against acute carbon monoxide [62] poisoning and reduces delayed neurologic sequelae [63].

### Hydrogen therapy and radiation injury

Radiotherapy is an important modality of cancer treatment, but ionizing radiation-induced damage caused by · OH following radiolysis of H_2_O is a problem and is usually initially dealt with by emergency department and intensive care unit physicians. It has been reported that HRS has a cardioprotective effect against radiation-induced injury [46]. Qian et al. [64] demonstrated that intraperitoneal injection of HRS before irradiation protected the gastrointestinal endothelia from radiation-induced injury. Radiation pneumonitis is another common complication. Chuai and colleagues reported that aerosol inhalation of a hydrogen-rich solution may be an effective and novel preventive strategy for radiation pneumonitis [65]. Hydrogen also had a radioprotective effect in vivo [66]. In addition, hydrogen preserved spermatogenesis and hematopoiesis [67] and protected against immune dysfunction [68].

### Methods of hydrogen intake

**Figure 2:**
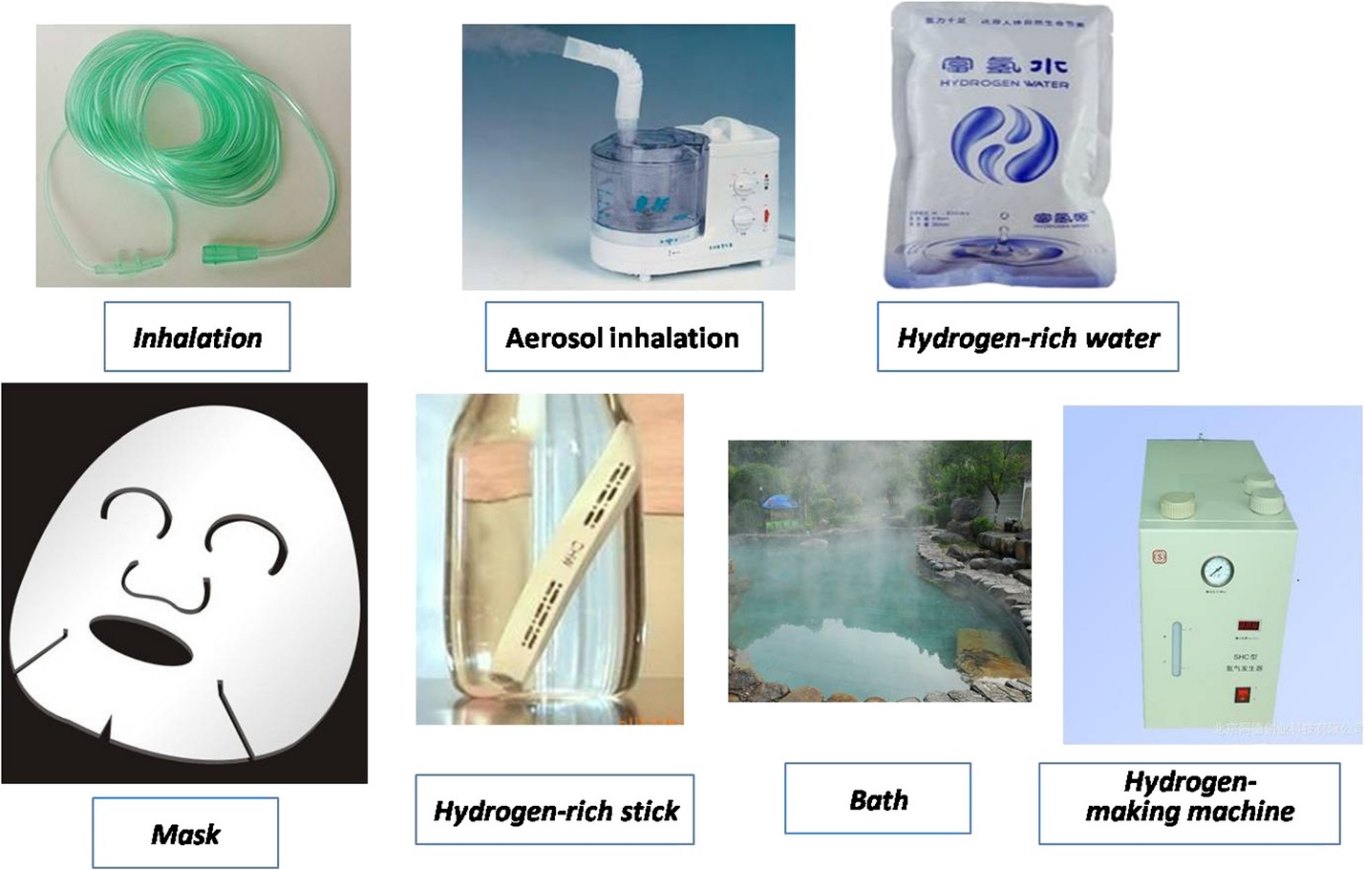
**All kinds of methods of hydrogen intake.**

There are many kinds of methods to take in hydrogen (Figure 2). The main means by which hydrogen may enter the body are breathing, drinking water, injection of saturated salt water, diffusion through the skin and endogenous hydrogen production by *Escherichia coli*. Hydrogen gas can be easily delivered via inhalation through a ventilator circuit, facemask or nasal cannula and is a straightforward therapeutic option. Hydrogen poses no risk of explosion in air or in pure oxygen when present at concentrations below 4%. However, safety remains a concern and the concentration of hydrogen must be monitored and maintained at the desired level with proven, commercially available tools [69]. The safety of hydrogen for humans is demonstrated by its use in hydreliox, an exotic breathing gas mixture of 49% hydrogen, 50% helium and 1% oxygen that is used for prevention of decompression sickness and nitrogen narcosis during very deep sea diving [70]. In air, hydrogen is present at a volume fraction of 4–75% and may even reach combustible levels; however, if the temperature does not exceed 500°C the gas will not burn, so the risk is controllable.

Solubilized hydrogen is a portable, easily administered and safe means of delivering molecular hydrogen [71]. Drinking hydrogen-rich water has an effect comparable to that of hydrogen inhalation [56]. Hydrogen water can be made by several methods, included dissolving electrolyzed hydrogen in pure water, dissolving hydrogen in water under high pressure and by the use of a hydrogen gas releasing agent (magnesium reacted with water: Mg +2H_2_O → Mg(OH)_2_ + H_2_). Hydrogen water is also produced and sold commercially. Administration of hydrogen via an injectable hydrogen-rich vehicle may allow the delivery of more accurate concentrations. In the future “hydrogen bath” services will become available, and through nanoreactor engineering we may improve hydrogen release [72].

### Future directions

**Figure 3:**
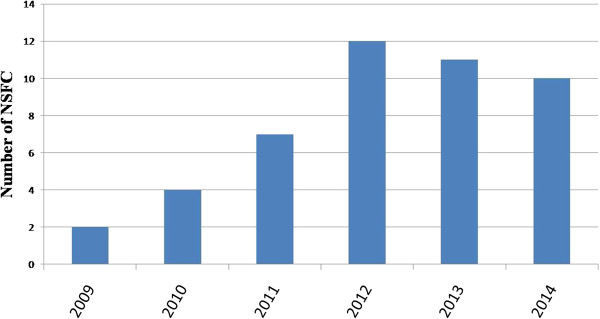
**The number of NSFC associated with molecular hydrogen in each year.**

Use of hydrogen for preventive and therapeutic purposes is a new field of investigation, for example, just in China there are 45 National Natural Science Foundation of China (NSFC) from 2009 to 2014 (Figure 3). Thus, there is limited information available on the pathways and processes that are influenced in vivo. Given the reported data, no other mechanism has been found to replace the reduction of ∙OH by hydrogen. Further studies are needed to elucidate the precise mechanism and signaling pathway involved in the protective role of hydrogen as a biological molecule. It has been proposed that hydrogen may act as a gaseous signaling molecule like nitric oxide, carbon monoxide or hydrogen sulfide [73]. This new viewpoint needs further investigation. At the time of writing, only about 10 papers concerning clinical work have been published [74]. More randomized, placebo controlled trials to optimize the dose, timing and delivery of hydrogen are needed. In addition, because of its extensive and diverse effects, hydrogen seems to differ from conventional drugs that act specifically on their pharmacologic targets, the pharmacokinetics, biology and toxicity of hydrogen remains incompletely understood.

## Conclusions

Hydrogen is a new potential therapeutic agent for the treatment of various diseases in emergency and critical care medicine. Although further investigations are required, with deepening of the basic theory and research into clinical applications, hydrogen may meet medical needs that at present incur considerable health burdens.
